# Berberine alleviates concanavalin A–induced autoimmune hepatitis in mice by modulating the gut microbiota

**DOI:** 10.1097/HC9.0000000000000381

**Published:** 2024-03-11

**Authors:** Hao Yang, Qingqing Liu, Haixia Liu, Xing Kang, Haixia Tian, Yongbo Kang, Lin Li, Xiaodan Yang, Peng Ren, Xiaoyu Kuang, Xiaohui Wang, Linzhi Guo, Mingwei Tong, Jieqiong Ma, Weiping Fan

**Affiliations:** 1 Department of Microbiology and Immunology, Shanxi Medical University, Jinzhong, China; 2 Key Laboratory of Cellular Physiology (Shanxi Medical University), Ministry of Education, and Shanxi Key Laboratory of Cellular Physiology, Taiyuan, China; 3 Laboratory of Morphology, Shanxi Medical University, Jinzhong 030619, China

## Abstract

**Background:**

Autoimmune hepatitis (AIH) is an immune-mediated liver disease of unknown etiology accompanied by intestinal dysbiosis and a damaged intestinal barrier. Berberine (BBR) is a traditional antibacterial medicine that has a variety of pharmacological properties. It has been reported that BBR alleviates AIH, but relevant mechanisms remain to be fully explored.

**Methods:**

BBR was orally administered at doses of 100 mg⋅kg^−1^⋅d^−1^ for 7 days to mice before concanavalin A–induced AIH model establishment. Histopathological, immunohistochemical, immunofluorescence, western blotting, ELISA, 16S rRNA analysis, flow cytometry, real-time quantitative PCR, and fecal microbiota transplantation studies were performed to ascertain BBR effects and mechanisms in AIH mice.

**Results:**

We found that liver necrosis and apoptosis were decreased upon BBR administration; the levels of serum transaminase, serum lipopolysaccharide, liver proinflammatory factors TNF-α, interferon-γ, IL-1β, and IL-17A, and the proportion of Th17 cells in spleen cells were all reduced, while the anti-inflammatory factor IL-10 and regulatory T cell proportions were increased. Moreover, BBR treatment increased beneficial and reduced harmful bacteria in the gut. BBR also strengthened ileal barrier function by increasing the expression of the tight junction proteins zonula occludens-1 and occludin, thereby blocking lipopolysaccharide translocation, preventing lipopolysaccharide/toll-like receptor 4 (TLR4)/ NF-κB pathway activation, and inhibiting inflammatory factor production in the liver. Fecal microbiota transplantation from BBR to model mice also showed that BBR potentially alleviated AIH by altering the gut microbiota.

**Conclusions:**

BBR alleviated concanavalin A–induced AIH by modulating the gut microbiota and related immune regulation. These results shed more light on potential BBR therapeutic strategies for AIH.

## INTRODUCTION

Autoimmune hepatitis (AIH) is a progressive immune-mediated liver disease that can lead to cirrhosis, HCC, and death, while its underlying mechanisms remain unclear.^1^,^2^ Patients with AIH usually receive long-term glucocorticoid treatment alone or with other immunosuppressive therapies, with most responding well to glucocorticoids, but serious side effects such as immunosuppression, secondary infection, abnormal increases in blood pressure and blood sugar, and osteoporosis mean that most patients relapse after drug withdrawal. Therefore, developing new prevention and treatment methods to reduce morbidity and mortality is required.^3^,^4^

Recently, reciprocal interactions between the liver and gut, termed the gut-liver axis, have garnered intense research activity.^5^ Alterations to the gut microbiota, such as decreases in microbial diversity, the depletion of beneficial bacteria, and the enrichment of opportunistic pathogens, are frequently reported in liver diseases.^6^ Intestinal dysbiosis has already been observed in patients with AIH; it was previously reported that the fecal microbiome of patients with AIH exhibited decreased species richness and evenness when compared with healthy controls.^7^ These findings suggested that modulating gut microbiota structures may be a useful scientific basis for AIH treatment.

Berberine (BBR) is an isoquinoline alkaloid extracted from herbal plants such as *Coptis chinensis* and *Berberis vulgaris*, and is traditionally used to treat bacterial diarrhea in China.^8^ BBR displays potent beneficial effects toward NAFLD by reducing lipid accumulation by activating AMP-activated protein kinase signaling, alleviating oxidative stress, and regulating the gut microenvironment.^9^ Wang et al^10^ showed that BBR pretreatment reduced liver injury in concanavalin A (Con A)–induced AIH mice via AMP-activated protein kinase signaling activation. Interestingly, the gut microbiota was recently recognized as an important target of BBR,^11^,^12^ with studies reporting that BBR stimulated *Akkermansia muciniphila* growth via enhanced mucin production in host intestines.^11^ However, it remains unknown whether BBR improves AIH by regulating intestinal microbiology and associated immune regulation. The mechanisms whereby BBR alleviates AIH require further exploration, thus elucidating such mechanisms may provide safer, more effective, and more affordable alternatives for treating AIH.

In this study, we observed that BBR alleviated Con A–induced AIH by altering the gut microbiota, inhibiting lipopolysaccharide (LPS)/toll-like receptor 4 (TLR4)/NF-κB signaling, and modulating the regulatory T cell (Treg)/Th17 balance. Additionally, the effects of BBR on AIH may be partly dependent on the changes in intestinal flora composition.

## METHODS

### Reagents

We used the following materials: Con A was acquired from Sigma-Aldrich (St. Louis, MO). BBR, alcian blue stain kit, methylprednisolone (MP), ampicillin, neomycin, metronidazole, and vancomycin were afforded by Solarbio (Beijing, China). Anti-Caspase-3, anti-Bax, and anti-F4/80 antibodies were offered by cell signaling technology (Boston, MA). Anti-TNF-α, anti-interferon-γ, anti-IL-1β, anti-IL-17A, and anti-IL-10 antibodies were purchased from Bioss (Beijing, China). Rabbit anti-mouse primary antibodies, including zonula occludens-1 (ZO-1), occludin, TLR4, NF-κB P65, inhibitor of NF-κB (IκB), phospho-inhibitor of NF-κB (p-IκB), and β-actin, were obtained from Abcam (Cambridge, UK). Allophycocyanin-conjugated anti-CD4, fluorescein isothiocyanate–conjugated anti-IL-17A, and phycoerythrin-conjugated anti-Foxp3 antibodies were purchased from BD (Franklin Lakes, NJ). LPS ELISA kits were supplied by Cloud-clone (Hubei, China).

### Experimental animals

Five-week-old male C57BL/6 mice weighting 18–20 g were obtained from the Experimental Animal Center of Shanxi Medical University (Shanxi, China). Animals were housed in a laminar flow, specific-pathogen–free facility under a 12-hour light/12-hour dark cycle with free access to a standard chow diet and water. Experimental procedures were performed based on the guidelines of the Regulation of the Administration of Laboratory Animals and approved by the Animal Experiment Ethics Committee of Shanxi Medical University (Permit Number: SYDL2021001).

### BBR interventions

**FIGURE 1:**
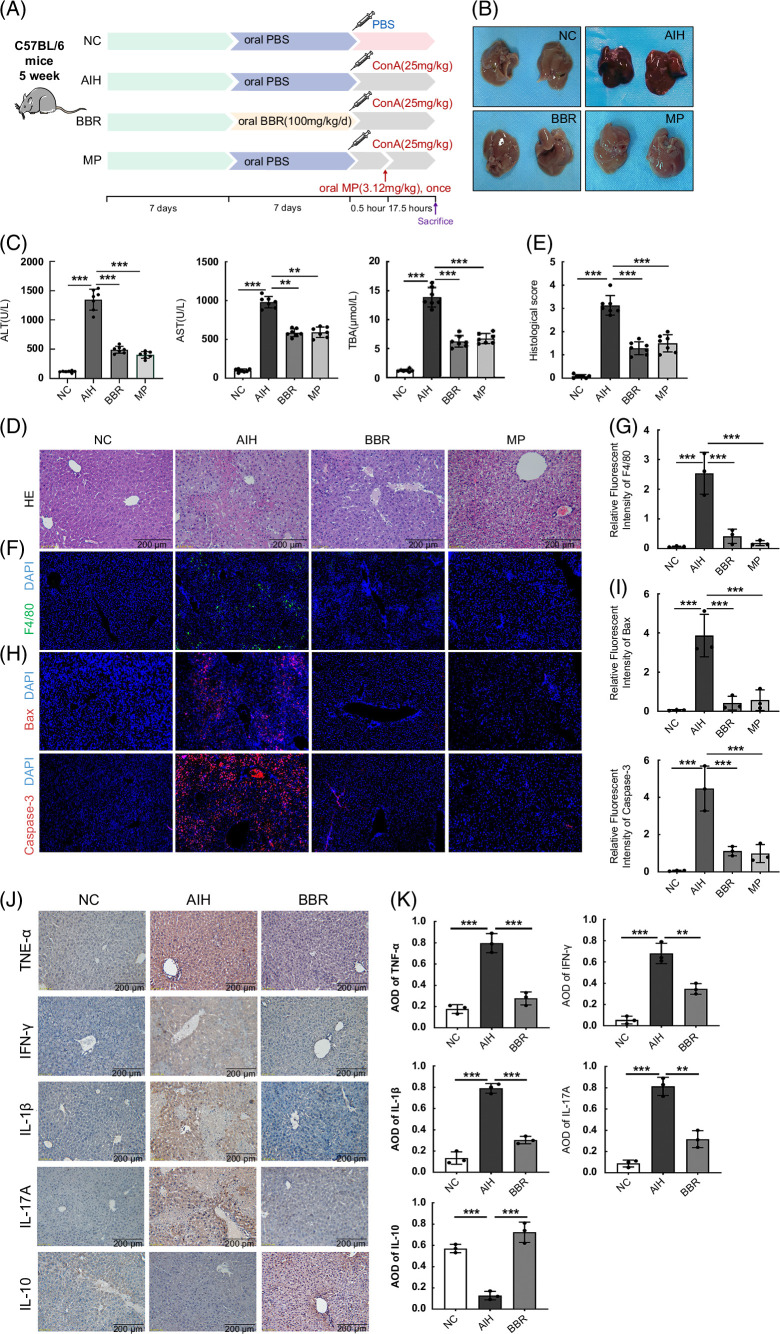
BBR alleviated Con A–induced liver injury. (A) Design of the animal experiment, (B) Macroscopic appearance of livers, (C) Concentration of ALT, AST, and TBA (n = 7), (D, E) HE staining of liver sections (scale bar, 200 µm) and the histological scores for liver sections. (F, G) Immunofluorescence staining of the infiltration of macrophages (F4/80^+^) (Green) in liver tissue section, quantification of staining intensity of F4/80 in each group (n = 3). (H, I) Immunofluorescence staining of the expression level of Bax and Caspase-3 (Red) in liver tissue section, quantification of staining intensity of Bax and Caspase-3 in each group (n = 3). (J, K) The expression of TNF-α, IFN-γ, IL-1β, IL-17A, and IL-10 in the livers by immunohistochemistry (scale bar, 200 µm) (n = 3). All data were expressed as mean±SD. **p<0.01; ***p<0.001. Abbreviations: AIH, autoimmune hepatitis; ALT, alanine transaminase; AOD, average optical density; AST, aspartate transaminase; BBR, berberine; ConA, concanavalin A; HE, hematoxylin and eosin; IFN-γ, interferon-γ; MP, methylprednisolone; NC, negative control; TBA, total bile acid.

The mice were randomly assigned to 4 groups (n = 7 per group): the negative control (NC) group, the AIH group, the BBR group, and the MP group. After 7 days of acclimation, the mice in the BBR group were administered BBR (100 mg/kg/d) by oral gavage,^13^ while the mice in the other groups were given an equivalent volume of PBS. Pretreatments were continued for 7 days. Then, the mice in AIH, BBR, and MP groups were administered 25 mg/kg of Con A via the tail vein, while NC group mice were administered an equivalent PBS volume. The mice in the MP group received MP (3.12 mg/kg) by intragastric administration once at 0.5 hours after the Con A injection.^14^ All mice were then humanely sacrificed at 18 hours after Con A injections and tissues were gathered. Animal procedures are shown in Figure 1A.

### Fecal microbiota transplantation

The mice in a fecal microbiota transplantation (FMT) group were treated with ampicillin (1 mg/mL), neomycin (1 mg/mL), metronidazole (1 mg/mL), and vancomycin (1 mg/mL) in drinking water for 7 days prior to the administration of a fecal suspension from BBR mice.^15^,^16^ Specifically, 1 g of fecal sample from 6 BBR mice were homogenized in 5 mL of PBS, after which the mice in the FMT group were transplanted with 200 μL of the suspension by oral gavage once a day for 7 days.^17^,^18^ The mice in the antibiotics eliminate intestinal bacteria (ABX) group were treated with the same antibiotics in drinking water for 7 days, then given an equivalent PBS volume by oral gavage once a day. Subsequent treatments were the same as in BBR intervention experiments. The animal experiment procedure is shown in Figure 7A.

### Transaminase activity and endotoxin assay

After centrifuging at 1000*g* for 10 minutes, the concentrations of alanine transaminase (ALT), aspartate transaminase (AST), and total bile acids (TBAs) in the serum were measured using the automated chemistry analyzer (BioMajesty, Japan) from TaiYuan Hospital of Traditional Chinese Medicine. The serum LPS was measured using the ELISA kit according to the manufacturer’s guidelines (Cloud-clone, China).

### Histological evaluations

The liver and ileum tissue samples were fixed in 4% neutral buffered formalin solutions for 48 hours. Then the tissue samples were dehydrated with graded ethanol and embedded in paraffin wax. Tissue sections (4 μm thick) were cut onto glass slides and stored at room temperature. The paraffin wax–embedded tissue sections were then stained with hematoxylin and eosin and alcian blue for histological evaluation. To evaluate the degree of necrosis, an injury grading score system based on portal and intralobular inflammation, degeneration, and necrosis in the liver was conducted as reported.^19^,^20^ The extent of pathology was scored from 0 (no pathological change) to 4 (severe pathological change).

### Immunohistochemistry and immunofluorescence staining

After heating for 20 minutes at 60°C, liver or ileum paraffin sections were deparaffinized in xylene and rehydrated in decreasing ethanol concentrations, washed in PBS, and blocked in bovine serum albumin for 30 minutes at room temperature. For immunohistochemistry, liver sections were incubated overnight at 4°C with antibodies against TNF-α, interferon-γ, IL-1β, IL-17A, and IL-10. Liver sections were also incubated with biotin-conjugated secondary antibodies at room temperature for 60 minutes and stained with 3, 3′-diaminobenzidine. Images were obtained using an Olympus upright microscope (Olympus Corporation, Japan), and Image J software was used to calculate average optical density.

For immunofluorescence (IF) staining, liver sections were stained with primary anti-F4/80 antibody, anti-Bax antibody, or anti-Caspase-3 antibody at 4°C overnight, incubated with the corresponding secondary antibodies for 60 minutes at room temperature the next day, and underwent a DAPI reaction. Ileum sections were stained with primary anti-ZO-1 antibody or anti-occludin antibody at 4°C overnight and incubated with the corresponding secondary antibodies for 60 minutes at room temperature. Cell nuclei were stained with DAPI. Then it was observed under a fluorescence microscope (Nikon, Japan).

### Flow cytometry

Detailed operational steps for this procedure are outlined in our previous research.^21^ Briefly, after animals were humanely sacrificed, spleen cell suspensions were prepared and cell concentrations adjusted to 1.0×10^7^/mL. To detect Th17 and Treg cells, 1 mL of single cells were seeded into wells in 6-well plates and incubated with ionomycin, phorbol-12-myristate-13-acetate, and Brefeldin A for 5 hours and harvested. Then, harvested cells were stained with corresponding antibodies. Samples were processed on a BD Accuri C6, and data were analyzed in FlowJo 7.6 analysis software (BD Biosciences, USA).

### RNA extraction and real-time quantitative PCR

Total RNA was isolated from fresh ileum tissue using Trizol reagent as per manufacturer’s instructions. Then, cDNA was synthesized from an equal amount of total RNA according to the manufacturer’s instructions. The products were mixed with SYBR Green PCR mix and subjected to real-time quantitative PCR using the BIOER LineGene 9600 Plus Fluorescent Quantitative Detection System (FQD-96A, BIOER, China). The primers used in real-time quantitative PCR are shown in Table 1.

**TABLE 1:** Primer sequences used for real-time quantitative PCR analysis

Mice gene	Forward (5′-3′)	Reverse (5′-3′)
β-actin	ATCAGCAAGCAGGAGTATG	GGTAGAGGACCACTTTGCTA
ZO-1	GCGAACAGAAGGAGCGAGAAGAG	GCTTTGCGGGCTGACTGGAG
Occludin	TGGACTTGGAGGCGGCTATGG	AGGGAAGCGATGAAGCAGAAGGC

Abbreviation: ZO-1, zonula occludens-1.

### Western blotting

Detailed operational steps for this procedure are outlined in our previous research.^21^ The protein bands were visualized using an enhanced chemiluminescence kit (Solarbio, Beijing, China). Band intensities were quantitatively analyzed using the ImageJ software, and protein expression was normalized to β-actin signals.

### Fecal 16S rRNA analysis

Cecal content samples were freshly collected in germ-free tubes and immediately frozen with liquid nitrogen, then stored at −80°C. DNA was extracted from feces using the QIAamp DNA Stool Mini Kit (Qiagen, USA). Sequences with ≥97% similarity were assigned to the same operational taxonomic units. The fecal microbial composition was assessed using Illumina HiSeq sequencing and QIIME-based microbiota analysis. The Wilcoxon rank sum test was performed to evaluate alpha diversity and beta diversity between the different cohorts in the 16S sequencing analysis. Correlations were tested for significance by the Spearman rank correlation test. The datasets generated for this study can be found in the NCBI Sequence Read Archive database (accession number: PRJNA789741).

### Statistical analysis

The data are expressed as the mean± SD, and the Shapiro-Wilk test was used to check for normality. For most data, one-way ANOVA was used to determine the significance between the groups. Statistical analyses were performed using GraphPad Prism 9.2.0 (GraphPad Software, USA) and SPSS version 22.0 software (SPSS Inc., USA). A *p*-value <0.05 was considered statistically significant.

## RESULTS

### BBR alleviates Con A–induced liver injury

To investigate the effects of BBR on AIH pretreatment, we established an AIH mouse model using Con A injections. Significantly elevated serum ALT, AST, and TBA were detected in the AIH group (*p*<0.001). When compared with the AIH group, both BBR and MP groups had significantly reduced serum ALT, AST, and TBA concentrations (*p*<0.01, *p*<0.001) (Figure 1C). Morphological observations are shown (Figure 1B). Macroscopically, BBR and MP administration ameliorated passive hepatic congestion induced by Con A. The hematoxylin and eosin staining of liver sections showed an extensive loss of liver architecture and hepatocyte necrosis in the AIH group, while BBR alleviated this liver injury (Figure 1D). Although MP also alleviated this liver injury, liver cell edema was observed in this group (Figure 1D, E), which revealed that BBR ameliorated AIH-induced pathological damage in mice and showed the same, if not better effects than MP.

Macrophages are inflammatory cells that exhibit strong phagocytosis activity and release many inflammatory factors that promote liver inflammation and damage.^22^ IF showed a markedly increased macrophage (F4/80+) infiltration in the AIH group when compared with the NC group, while macrophage fluorescence intensity was significantly decreased in BBR and MP groups (Figure 1F, G). Bax and Caspase-3 fluorescence intensity levels were both significantly enhanced in the livers of the AIH group when compared with the NC group, while fluorescence intensity was markedly decreased in BBR and MP groups (Figure 1H, I). Immunohistochemistry results showed that TNF-α, interferon-γ, IL-1β, and IL-17A expression levels in the liver were markedly increased in the AIH group when compared with NC and BBR groups. In addition, BBR administration increased levels of the anti-inflammatory cytokine IL-10 in the liver (Figure 1J, K), which indicated that BBR administration inhibited proinflammatory cytokine production and promoted IL-10 production in our Con A–induced AIH mouse model. Taken together, these results demonstrated that BBR ameliorated Con A–induced acute liver injury by reducing hepatocyte inflammation, necrosis, and apoptosis.

### The immunoregulatory effects of BBR administration in AIH mice

**FIGURE 2:**
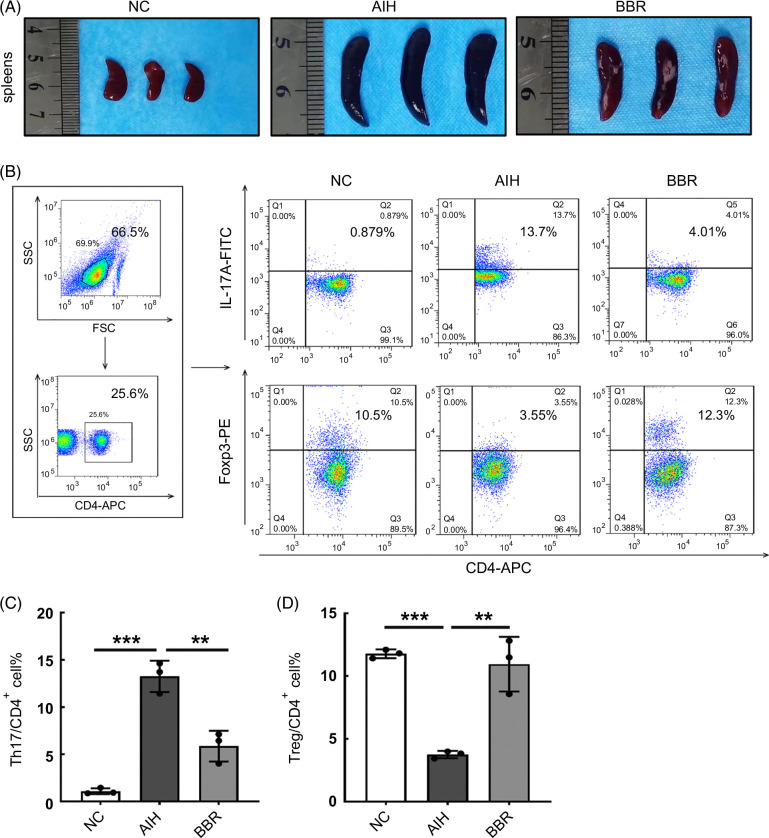
BBR restored immune imbalance induced by concanavalin A in mice. (A) Macroscopic appearance of spleens. (B) Typical flow cytometry plot of Th17 cells and Treg cells in the spleens in NC, AIH, and BBR groups of mice (n = 3). (C, D) Bar charts showing the percentages of Th17 cells and Treg cells in CD4^+^T cells (n = 3). All data were expressed as mean±SD. **p<0.01; ***p<0.001. Abbreviations: AIH, autoimmune hepatitis; APC, allophycocyanin; BBR, berberine; FITC, fluorescein isothiocyanate; FSC, forward scatter; NC, negative control; SSC, side scatter.

As shown (Figure 2A), spleens from the mice in the AIH group were significantly swollen when compared with those in the NC group, while spleens in the BBR group were smaller than those in the AIH group, which indicated that BBR mice were in a lower inflammatory state when compared with the AIH group. The Th17 and Treg cell balance regulates the development of autoimmunity and inflammation and is considered a potential therapeutic target for liver injury.^23^ We next evaluated Th17 and Treg cell percentages in mouse spleens by flow cytometry (Figure 2B). When compared with the NC group, Th17 cell percentages were immediately increased, while Treg cell percentages were decreased in the AIH group (*p*<0.001). When compared with the AIH group, Th17 cell percentages decreased while Treg cell percentages increased in the BBR group (*p*<0.01) (Figure 2C, D). Based on these data, BBR administration exerted immunosuppressive effects in our Con A–induced AIH model.

### BBR reinforces intestinal barrier function

**FIGURE 3:**
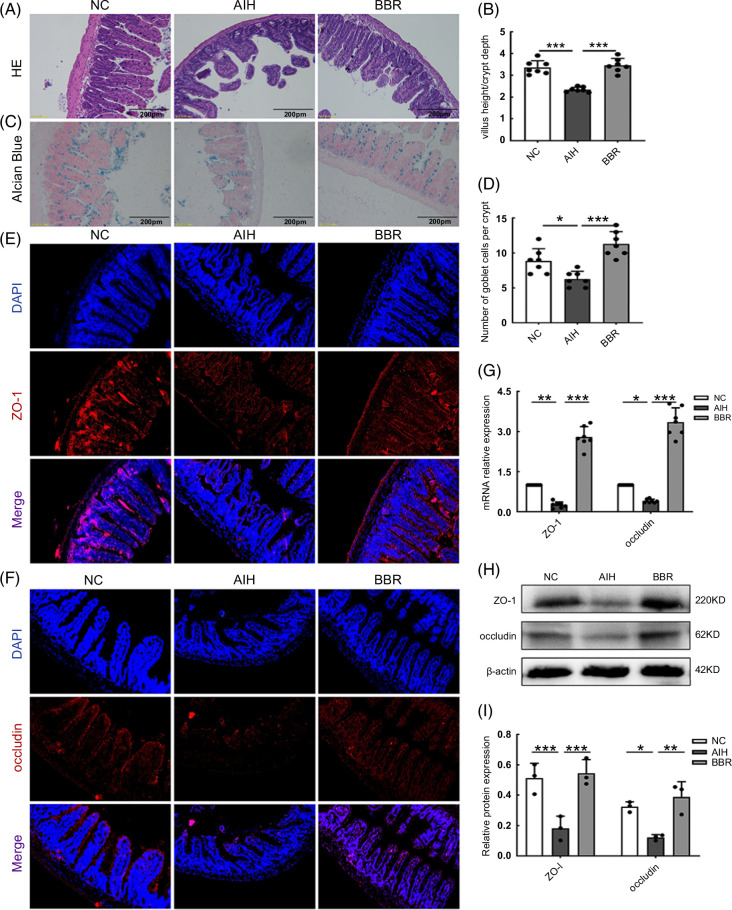
BBR improved intestinal barrier function. (A) HE staining of ileum sections (scale bar, 200 µm). (B) Five crypts per section were evaluated, then microscopically assessed villus height and crypt depth and calculated the ratio of villus height and crypt depth (n = 7). (C) Alcian blue staining of ileum sections (scale bar, 200 µm). (D) The number of acid-mucin–producing goblet cells per villus (n = 7). (E, F) The membrane localization of ZO-1 and occludin (red) was assessed by immunofluorescence and visualized by fluorescence microscopy, nuclei were stained with DAPI (blue) (n = 3). (G) Total RNA was extracted from the ileum tissues for real-time quantitative PCR analysis. The relative expressions of ZO-1 and occludin were shown (n = 7). (H, I) ZO-1 and occludin expressions in the ileum tissues from each group were evaluated by western blotting and the relative protein expression was quantified (n = 3). All data were expressed as mean±SD. *p<0.05; **p<0.01; ***p<0.001. Abbreviations: AIH, autoimmune hepatitis; BBR, berberine; HE, hematoxylin and eosin; NC, negative control; ZO-1, zonula occludens-1.

Impaired intestinal barrier function may increase the risk of bacterial component access from the gut lumen to the bloodstream, thereby aggravating systemic inflammation.^24^ Histopathological examinations revealed that the ileum exhibited histological abnormalities after the Con A challenge. As shown (Figure 3A), epithelium villi and ileum crypts in the NC group were tightly and neatly attached; villous rupture and denuded states were observed in the AIH group, while BBR administration alleviated intestinal epithelial damage caused by Con A.

We next evaluated intestinal morphological alterations by calculating villus height to crypt depth ratios. As shown (Figure 3B), ratios in the AIH group were significantly decreased when compared with the NC group, while BBR restored ratios back to NC levels (*p*<0.001) (Figure 3B). From Alcian blue staining of the ileum, we observed a marked reduction in goblet cell (GC) numbers in the AIH group when compared with the NC group, which were normalized by BBR (*p*<0.05 and <0.001) (Figure 3C, D).

Levels of the tight junction (TJ) proteins, namely ZO-1 and occludin, were determined by real-time quantitative PCR, western blotting, and IF staining. In contrast with the AIH group, NC and BBR groups had higher ZO-1 and occludin expression levels and a uniform positive distribution of both proteins at the apical region of the ileum (Figure 3E, F). We observed that ZO-1 and occludin mRNA expression levels in ileum tissue were significantly higher in the BBR group when compared with the AIH group (*p*<0.001) (Figure 3G). ZO-1 and occludin protein expression levels in ileum tissue were also increased in the BBR group when compared with the AIH group (*p*<0.01 and <0.001) (Figure 3H, I). These results showed that Con A–induced ZO-1 and occludin expression loss was prevented when mice were administered BBR.

### BBR inhibits LPS translocation and TLR4/NF-κB signaling activation

**FIGURE 4:**
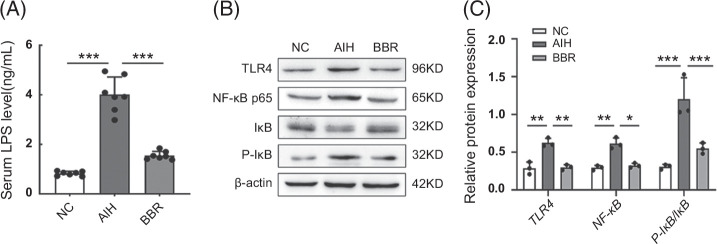
BBR inhibited LPS translocation and activated the TLR4/NF-κB signaling pathway in the liver of AIH mice. (A) Serum concentration of LPS in each group were detected by ELISA (n = 7). (B) TLR4, NF-κB, IκB, and P-IκB expressions in the liver tissues from each group were evaluated by western blotting (n = 3). (C) The relative protein expressions of TLR4, NF-κB, and P-IκB/IκB were quantified (n = 3). All data were expressed as mean±SD. *p<0.05; **p<0.01; ***p<0.001. Abbreviations: AIH, autoimmune hepatitis; BBR, berberine; LPS, lipopolysaccharide; NC, negative control; p-IκB, phospho-inhibitor of NF-κB; TLR4, toll-like receptor 4.

Plasma endotoxins, which are intestinal barrier function markers, are key elements that indicate liver damage progression.^25^ When the intestinal barrier is damaged, bacterial components, represented by LPS, break through the barrier and may cause serious liver injury.^26^ We observed that serum LPS concentrations and TLR4, NF-κB, and P-IκB/IκB expression levels in the liver were markedly decreased in the BBR group when compared with the AIH group (*p*<0.001, <0.05, <0.01, or <0.001) (Figure 4A–C). Thus, BBR administration inhibited LPS translocation by strengthening the intestinal barrier and suppressing TLR4/NF-κB signaling activation. These results also provided extra evidence of strengthened intestinal barrier function upon BBR administration.

### BBR alters gut microbiota structure in AIH mice

**FIGURE 5:**
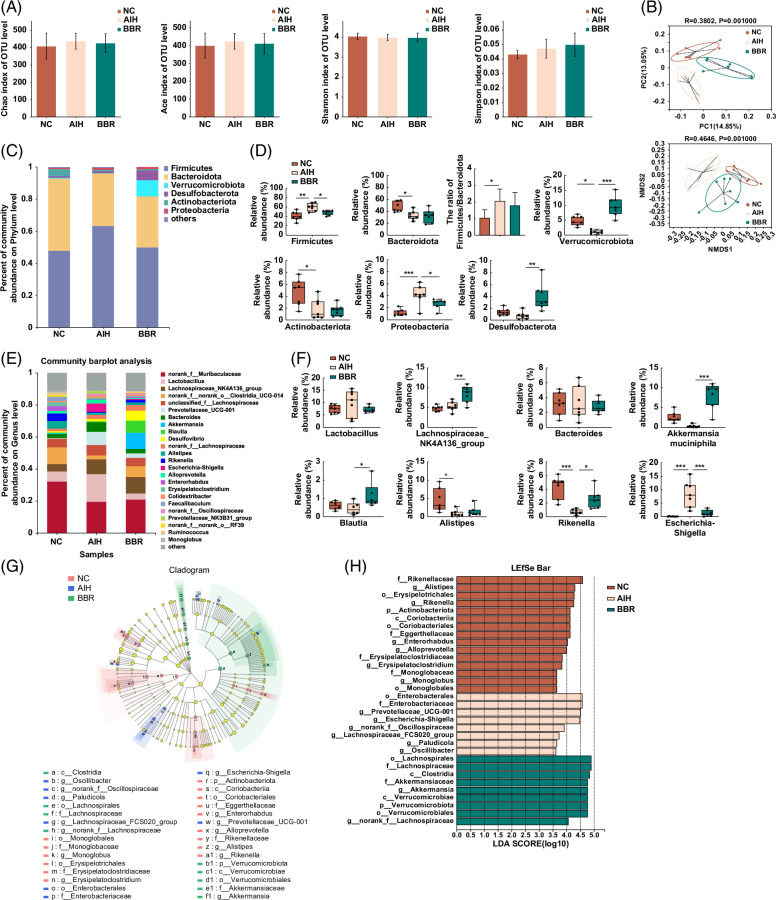
BBR changed the structure of gut microbiota in Con A–induced AIH mice. Total fecal bacteria from each mouse were detected by 16S rRNA sequencing. (A) α-diversity estimated by Chao, Ace, Shannon, and Simpson indices. (B) The Principal Coordinate Analysis (PCoA) and nonmetric multidimensional scaling (NMDS) plot showing the β-diversity of the gut microbiota between the three groups based on the unweighted UniFrac metric. Each symbol represents one sample. (C) Community barplot analysis of each group on phylum level. (D) The relative abundance of Firmicutes, Bacteroidota, Verrucomicrobia, Desulfobacterota, Actinobacteriota, and Proteobacteria at the phylum level. (E) Community barplot analysis of each group on genus level. (F) The relative abundance of *Lactobacillus*, *Lachnospiraceae_NK4A136_group*, *Bacteroides*, *Akkermansia muciniphila*, *Blautia*, *Alistipes*, *Rikenella*, and *Escherichia-Shigella* at the genus level. (G) LEfSe cladograms represented taxa enriched in NC group (Red), AIH group (Blue), and BBR group (Green). (H) Gut microbiota comparisons among the three groups analyzed by LEfSe at different taxonomy levels (LDA score >3.5). Data were expressed as mean±SD. n =7 per group, *p<0.05; **p<0.01; ***p<0.001. Abbreviations: AIH, autoimmune hepatitis; BBR, berberine; LDA, linear discriminant analysis; LEfSe, linear discriminant analysis effect-size method; NC, negative control; NMDS, nonmetric multidimensional scaling; OUT, operational taxonomic unit.

To investigate the influence of BBR on gut microbiota composition, cecal contents were assessed by 16S rRNA sequencing. Alpha diversity, as represented by Chao, Ace, Shannon, and Simpson indices, showed no significant differences between the 3 groups, indicating that overall microbial diversity and richness were similar (Figure 5A). However, Con A injections distinctly altered β-diversity in the gut microbiota of mice. Unweighted Unifrac Principle Component Analysis and nonmetric multidimensional scaling analysis, based on operational taxonomic units, revealed that gut microbiota in the AIH group had separated from the NC group, and that gut microbial community structures had also segregated differently between groups (Figure 5B).

Operational taxonomic unit analyses revealed the relative abundance of gut microbiota at phylum and genus classification levels. At the phylum level, gut microbiota composition in groups is shown (Figure 5C). In all samples, the 2 most dominant phyla were *Firmicutes* and *Bacteroidota*. Interestingly, the relationship between these 2 dominant phyla, expressed as the *Firmicutes*/*Bacteroidota* ratio, has been associated with several pathological conditions.^27^ As shown (Figure 5D), Con A injections increased the *Firmicutes*/*Bacteroidota* ratio in the AIH group more so than the NC group (*p*<0.05). When compared with the NC group, the relative abundance of *Firmicutes* and *Proteobacteria* in the AIH group increased significantly, while the relative abundance of *Bacteroidota*, *Actinobacteriota*, and *Verrucomicrobia* decreased significantly (*p*<0.001, <0.01, or <0.05). When compared with the AIH group, the relative abundance of *Firmicutes* and *Proteobacteria* decreased in the BBR group, while the relative abundance of *Verrucomicrobia* and *Desulfobacterota* increased significantly (*p*<0.001, <0.01, or <0.05).

At the genus level, gut microbiota composition in groups is shown (Figure 5E). When compared with the NC group, the relative abundance of *Escherichia-Shigella* increased significantly in the AIH group, while the relative abundance of *Alistipes* and *Rikenella* decreased significantly (*p*<0.001 or <0.05). When compared with the AIH group, the relative abundance of *Escherichia-Shigella* decreased in the BBR group, while the relative abundance of *Lachnospiraceae_NK4A136_group*, *Akkermansia muciniphila*, *Blautia*, and *Rikenella* increased significantly (*p*<0.001, <0.01, or <0.05) (Figure 5F).

Moreover, the linear discriminant analysis effect-size method (LEfSe) was used to discriminate biomarkers and dominant microbiota in groups (linear discriminant analysis >3.5, *p*<0.05). As shown (Figure 5H), at the family level, LEfSe analysis showed that *Rikenallaceae* were enriched in the NC group, *Enterobacteriaceae* were enriched in the AIH group, and *Lachnospiraceae* and *Akkermansiaceae* abundance was relatively higher in the BBR group when compared with other groups. At the genus level, LEfSe analysis showed that *Alistipes* and *Rikenella* were enriched in the NC group, *Prevotellaceae* and *Escherichia-Shigella* were enriched in the AIH group, and *A. muciniphila* abundance was relatively higher in the BBR group when compared with the other groups. As indicated (Figure 5G), key bacterial alterations are shown in the LEfSe taxonomic cladogram.

In summary, these results indicated that BBR administration appeared to modulate gut microbiota composition in AIH mice.

### Correlations between differential gut microbes and host parameters

**FIGURE 6:**
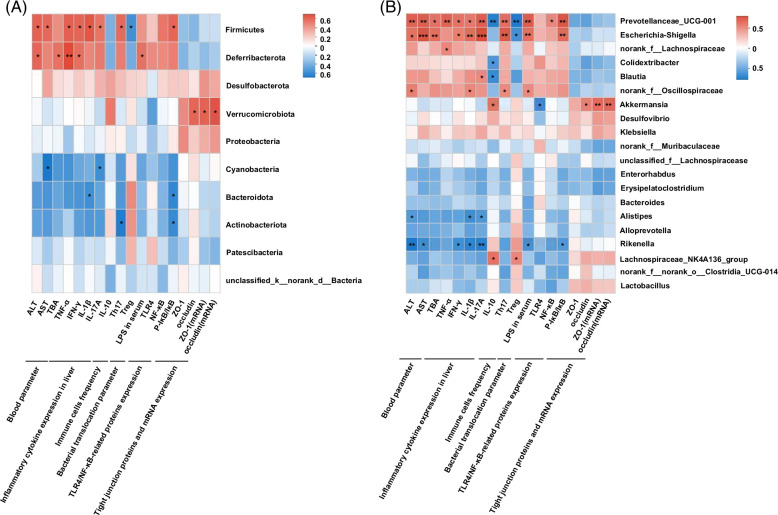
Spearman rank correlations between gut microbiota abundance and host parameters (A) on phylum level and (B) on genus level. Red indicate being positive correlation and blue being negative. *p<0.05; **p<0.01; ***p<0.001. Abbreviations: ALT, alanine transaminase; AST, aspartate transaminase; IFN-γ, interferon-γ; LPS, lipopolysaccharide; TBA, total bile acid; TLR4, toll-like receptor 4; ZO-1, zonula occludens-1.

We performed Spearman correlation analyses to evaluate potential links between significant changes in gut microbiota composition induced by BBR and host parameters, including liver injury indices, inflammatory cytokines, immune function indices, TJ proteins, and TLR4/NF-κB–related protein expression levels. Critically, these examinations further elucidated the relationships between different host parameters and microbial communities at phylum and genus levels. We observed that some microbes were closely associated with liver damage and inflammation. At the phylum level (Figure 6A), *Firmicutes* and *Deferribacterota* showed significant positive correlations between liver damage and inflammation. *Firmicutes* and *Actinobacteriota* showed a positive correlation with TLR4 expression. We also observed a significant negative correlation between serum AST, ALT, and TBA levels and gut microbiota abundance, of which *Cyanobacteria* showed the most significant negative correlation with AST levels. *Cyanobacteria* had the most significant negative correlation with IL-17A, while *Bacteroidota* demonstrated a significant negative correlation with IL-1β and P-IκB/IκB expressions. Notably, serum LPS concentrations were positively correlated with the relative abundance of *Deferribacterota*. The relative abundance of *Verrucomicrobiota* was positively associated with ZO-1 and occludin levels.

At the genus level (Figure 6B), we observed a significant negative correlation between AST, ALT, and TBA serum levels and gut microbiota abundance (in the top 20 genera). *Rikenella* showed the most significant negative correlation with ALT levels. It was important to note that *Prevotellaceae_UCG-001* and *Escherichia-Shigella* were positively correlated with hepatic injury markers, Th17 cell percentages, LPS concentrations, and P-IκB/IκB expression levels in livers. Intestinal barrier indices, including ZO-1 and occludin, were significantly positively correlated with the relative abundance of *A. muciniphila*, which was enriched in the BBR group. TLR4 expression in livers also showed a negative correlation with *A. muciniphila*. In addition, *Lachnospiraceae_NK4A136_group* was positively correlated with IL-10 expression in the liver and a proportion of Treg cells in the spleen. Therefore, we speculated that BBR modified the gut microbiota and played key roles in limiting the disadvantageous effects of Con A injections.

### The gut microbiota mediates susceptibility to AIH in mice

**FIGURE 7:**
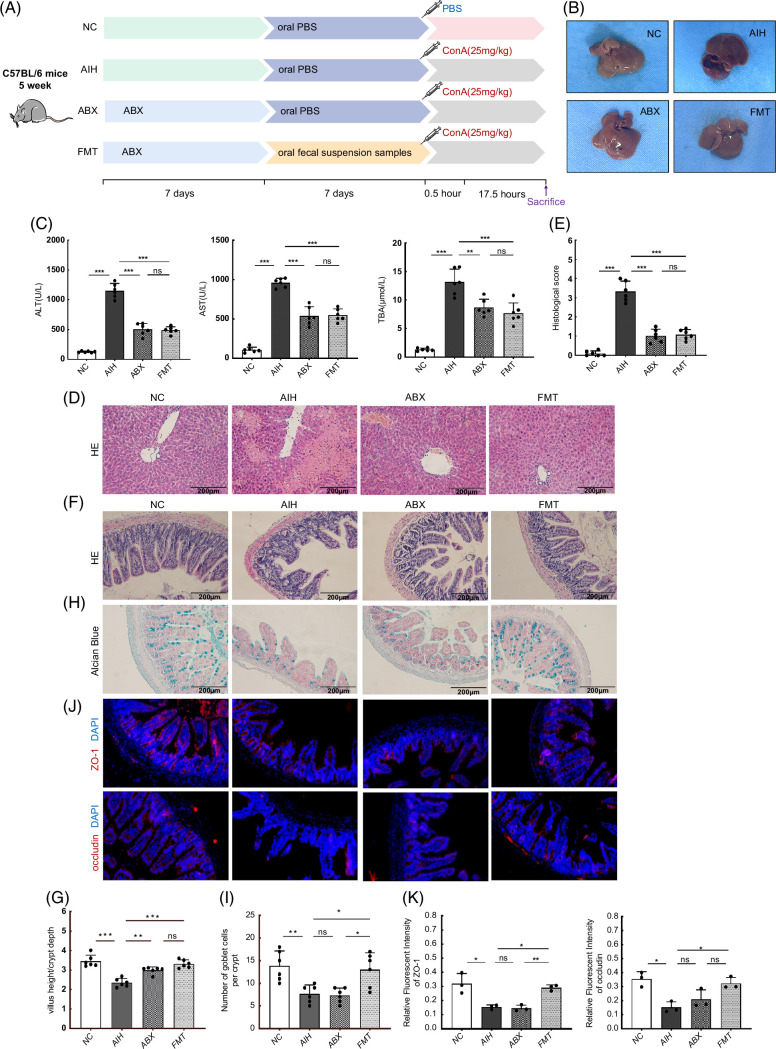
Gut microbiota is associated with ConA–induced liver injury, and FMT improved intestinal barrier function. (A) Design of the animal experiment. (B) Macroscopic appearance of livers. (C) Concentration of ALT, AST, and TBA (n = 6). (D, E) HE staining of liver sections (scale bar, 200 µm) and the histological scores for liver sections (n = 6). (F) HE staining of ileum sections (scale bar, 200 µm). (G) Five crypts per section were evaluated, then microscopically assessed villus height and crypt depth, and calculated the ratio of villus height and crypt depth (n = 6). (H) Alcian blue staining of ileum sections (scale bar, 200 µm). (I) The number of acid-mucin–producing goblet cells per villus (n = 6). (J) The membrane localization of ZO-1 and occludin (red) was assessed by immunofluorescence and visualized by fluorescence microscopy, nuclei were stained with DAPI (blue) (n = 3). (K) Quantification of staining intensity of ZO-1 and occludin in each group (n = 3). All data were expressed as mean±SD.*p<0.05; **p<0.01; ***p<0.001. Abbreviations: ABX, antibiotics eliminate intestinal bacteria; AIH, autoimmune hepatitis; ALT, alanine transaminase; AST, aspartate transaminase; ConA, concanavalin A; FMT, fecal microbiota transplantation; HE, hematoxylin and eosin; NC, negative control; ns, no significance; TBA, total bile acid; ZO-1, zonula occludens-1.

To investigate whether BBR reduced susceptibility to AIH in mice by regulating the gut microbiota, we conducted an FMT study and found that the mice transplanted with fecal samples from BBR mice were resistant to Con A–induced liver injury. We also observed that serum ALT, AST, and TBA levels in the mice in the FMT group were lower when compared with the levels in the AIH group (*p*<0.001) (Figure 7C). As shown (Figure 7B), liver morphology in the mice in the FMT group was similar to that in NC mice. Hematoxylin and eosin staining of liver sections showed that liver structures in FMT mice were normal with no hepatocyte necrosis (Figure 7D, E). We also observed that ABX mice had become resistant to Con A–induced liver injury after clearing intestinal commensal microbiota with antibiotics (Figure 7B–E). These findings demonstrated that the gut microbiota played important roles in AIH development.

Interestingly, intestinal epithelial damage caused by Con A was significantly alleviated in FMT mice after FMT from the mice in the BBR group. Specifically, villus height to crypt depth ratios and GC numbers in the ileum were both significantly increased in FMT mice when compared with the levels in the AIH group, factors critically important for intestinal barrier function (*p*<0.01 and <0.001) (Figure 7F–I). Furthermore, TJ proteins levels in groups were determined by IF staining. ZO-1 and occludin expression levels were increased in the ileum of FMT mice when compared with the levels in AIH mice (*p*<0.05) (Figure 7J, K). These results indicated that BBR had important roles inhibiting leaky guts and AIH development.

Lin et al^28^ reported that gut-derived LPS promoted Con A–induced liver injury in a TLR4-dependent manner, with LPS reductions mediated by antibiotic regimens markedly suppressing this liver injury. In our study, due to intestinal commensal microbiota deficiency in ABX mice, liver injury induced by Con A was significantly reversed. Interestingly, despite this reversal, intestinal barriers in ABX mice were similar to those in the AIH group. As shown (Figure 7G), villus height to crypt depth ratios in the ileum increased in ABX mice when compared with the AIH group (*p*<0.01). However, GC numbers and ZO-1 and occludin levels in the ileum were similar in both groups (*p*>0.05). These observations suggested that the gut microbiota had more important roles than intestinal barrier integrity in AIH development.

## DISCUSSION

Previous studies have shown that inflammatory infiltration and apoptosis in the liver are essential processes for inflammatory-related liver disease.^29^ In our study, we observed that Con A–induced serious hepatocyte architecture injury was effectively alleviated by BBR. Inflammatory cells, inflammatory factors, and the apoptosis-related proteins Bax and Caspase-3 were significantly decreased in livers upon BBR administration, while the anti-inflammatory cytokine IL-10 was increased.

Th17 cells secrete IL-17A, stimulating proinflammatory molecule secretion and further participating in autoimmune disease pathogenesis.^30^ Treg cells counterbalance tissue inflammation by secreting anti-inflammatory IL-10. Previous studies have demonstrated that BBR may inhibit Th17 responses in T-cell–mediated autoimmune diseases.^31^ Our study indicated that BBR improved the Treg/Th17 balance by raising Treg and lowering Th17 cell levels.

The intestinal epithelial barrier, which has evolved to maintain a balance between nutrient absorption and preventing toxin and luminal bacteria entry, consists of a mucus layer, epithelial cells, and intercellular junctions.^32^ TJ proteins comprise multifunctional complexes that seal paracellular spaces between epithelial cells, thus preventing the paracellular diffusion of microorganisms and other antigens across the epithelium.^33^ During barrier maintenance, GCs generate mucus layers that line the intestinal lumen.^34^ Normally, LPS transfer to extraintestinal organs is prevented by an intact intestinal barrier,^26^ but an impaired barrier may increase LPS translocation.^24^ Therefore, inhibiting LPS/TLR4/NF-κB signaling is key to relieving liver inflammation.^35^ Our results indicated that Con A injections destroyed the intestinal barrier, while BBR administration increased TJ protein expression, GC numbers, and improved ileum morphology. We also identified lower LPS serum levels and suppressed TLR4/NF-κB signaling in the BBR group, which as an influence of the reinforced intestinal barrier induced by BBR via a thickened mucus layer and enhanced TJs.

Gut microbiota disturbance may serve as a primary factor in augmenting LPS and proinflammatory cytokines in the lumen, which leads to increased intestinal permeability.^36^ The gut microbiota has been shown to be an important target of BBR.^11^,^37^ Our analyses showed that the gut microbiota participated in Con A–induced liver injury susceptibility, and that BBR administration appeared to beneficially reshape gut microbiota communities. Of note, among altered microbiota, *A. muciniphila* was the most highly influenced by BBR, consistent with observations by Dong et al.^11^ Therefore, significantly increased *A. muciniphila* abundance may improve intestinal barrier and immune functions and reduce LPS translocation.

Short-chain fatty acids (SCFAs) such as acetate, propionate, and butyrate are generated by the bacterial fermentation of dietary fiber in the intestinal lumen.^38^ Beyond their importance in intestinal integrity, SCFAs have potent anti-inflammatory effects. Previous studies have reported that SCFAs appear to regulate Treg cells to reduce inflammation.^39^ Treg cells maintain intestinal homeostasis by secreting anti-inflammatory cytokines, mainly IL-10, and inversely regulating Th17 cells.^40^ *Lachnospiraceae* is a probiotic that produces SCFAs.^41^ In our study, the *Lachnospiraceae_NK4A136_group* showed a positive correlation with the proportion of Treg cells in the spleen. In previous work, *Alistipes* demonstrated good anti-inflammatory effects in human and animal studies.^13^ In our study, *Alistipes* showed a negative correlation with IL-1β and IL-17A expressions in the liver. Thus, our results have provided additional evidence that BBR may reduce susceptibility to a Con A challenge by regulating the gut microbiota and the associated immune regulation events.

BBR has been proven to directly inhibit Con A–induced liver injury, so it cannot be ruled out that BBR directly attenuates liver injury, thereby improving gut microbiota and intestinal structures via gut-liver cross talk. To distinguish if gut microbiota alterations upon BBR intervention were the cause or consequence of AIH remission, we performed an FMT study. As hypothesized, we observed that tail vein injections of Con A in FMT mice did not cause severe liver injury, and that FMT also showed beneficial results in terms of intestinal-related indicators. These results suggested that the reshaped microbiota had improved AIH, and it was not a consequence of AIH remission. This further suggested that BBR mainly alleviated liver damage by improving intestinal microecology, enhancing intestinal function, reducing LPS hepatic translocation, and inhibiting the LPS/TLR4/NF-κB inflammatory pathway.

A previous study showed that liver injury in experimental AIH mice was significantly inhibited when intestinal commensal microbiota were cleared with broad-spectrum antibiotic mixtures,^42^ which suggested that S100-induced liver injury partly depended on intestinal commensal microbiota. Another study showed that when compared with specific-pathogen–free mice, Con A treatment did not trigger hepatic natural killer T cell activation in germ-free mice, which suggested that glucolipid antigens derived from intestinal commensal bacteria were necessary for natural killer T cell activation in Con A–induced liver injury.^43^ We also conducted similar studies and found that the ABX group showed significantly lower intestinal TJ protein expression, similar to the expression in AIH mice. However, liver damage was not obvious. These results suggested that the gut microbiota played more crucial roles in hepatitis occurrence, no matter if Con A or S100 was used to induce AIH. Thus, a commensal gut microbiome is required for innate immune response activation. However, gut microbiota roles in liver immunity remain controversial, and more studies are required to confirm this connection. A recent study on the relationship between acute pancreatitis and the gut microbiota in mice showed that when compared with specific-pathogen–free mice, the intestinal barrier in GF and ABX mice was disrupted, but acute pancreatitis severity was ameliorated due to a gut microbiota deficiency.^44^ This result was similar to ours and further indicated that increased intestinal permeability alone was not enough to cause intestinal-borne inflammation. Additionally, significantly decreased intestinal TJ protein expression in ABX mice also indicated that gut microbiota destruction resulted in low intestinal TJ protein expression.

BBR exhibited similar ameliorating effects as glucocorticoids on Con A–induced AIH but highlighted the effects of altering the gut microbiota and immune regulation. These findings suggested that BBR may represent a safer, more effective, and more affordable medicine for AIH treatment. Due to the effects of altering the gut microbiota and immune regulation, BBR may alleviate other liver diseases, such as viral hepatitis and hepatic fibrosis, via the gut-liver-immune axis. These findings may indicate a role for BBR in other autoimmune diseases. Exploration of these avenues may ultimately provide a wider range of uses for this traditional medicine.

## CONCLUSIONS

**FIGURE 8:**
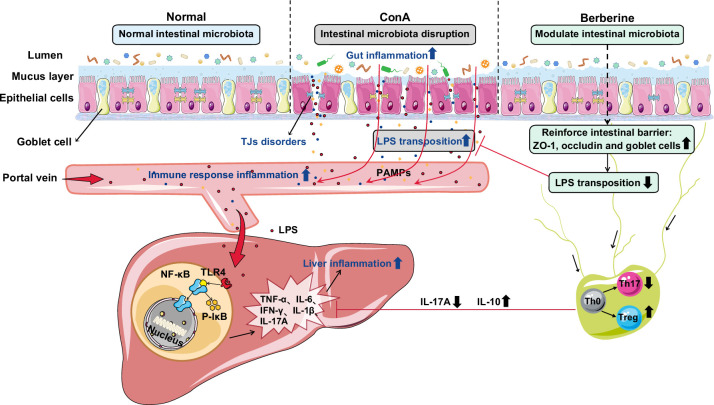
Protective mechanisms of berberine against autoimmune hepatitis mice. Abbreviations: ConA, concanavalin A; IFN-γ, interferon-γ; LPS, lipopolysaccharide; PAMPs, pathogen-associated molecular patterns; p-IκB, phospho-inhibitor of NF-κB; TJs, tight junctions; TLR4, toll-like receptor 4; ZO-1, zonula occludens-1.

Overall, we demonstrated that liver injury alleviation by BBR administration was related to an altered gut microbiota and a strengthened intestinal barrier. This latter observation inhibited the translocation of gut-derived and potentially harmful substances to the liver. Then, the LPS/TLR4/NF-κB signaling pathway was blocked, which led to reduced proinflammatory cytokine production. A reshaped gut microbiota may be related to a more beneficial Treg/Th17 balance and inflammatory cytokine expression. In summary, we propose that BBR targets the gut microbiota-immune system axis and may be used as a therapeutic agent in AIH therapy (Figure 8).
